# The Potential Roles of Very Low Calorie, Very Low Calorie Ketogenic Diets and Very Low Carbohydrate Diets on the Gut Microbiota Composition

**DOI:** 10.3389/fendo.2021.662591

**Published:** 2021-05-14

**Authors:** Mariangela Rondanelli, Clara Gasparri, Gabriella Peroni, Milena Anna Faliva, Maurizio Naso, Simone Perna, Philip Bazire, Ignacio Sajoux, Roberto Maugeri, Chiara Rigon

**Affiliations:** ^1^ IRCCS Mondino Foundation, Pavia, Italy; ^2^ Department of Public Health, Experimental and Forensic Medicine, Unit of Human and Clinical Nutrition, University of Pavia, Pavia, Italy; ^3^ Endocrinology and Nutrition Unit, Azienda di Servizi alla Persona “Istituto Santa Margherita”, University of Pavia, Pavia, Italy; ^4^ Department of Biology, College of Science, University of Bahrain, Sakhir, Bahrain; ^5^ PronoKal, London, United Kingdom; ^6^ PronoKal Group, Barcelona, Spain; ^7^ PronoKal Group, Savigliano, Italy

**Keywords:** very low carbohydrate diet (VLCarbD), very low calorie diet (VLCD), obesity, microbiota, very low calorie ketogenic diet (VLCKD), gut microbiota

## Abstract

Several studies have described a strong correlation between diet, weight loss, and gut microbiota composition. The aim of this review was to evaluate the potential effects of energy-restricted diets, namely very low calorie diets (VLCDs), very low calorie ketogenic diets (VLCKDs), and very low carbohydrate diets (VLCarbDs), on the composition of the gut microbiota in humans. We performed a literature search using the following terms (with their abbreviations or acronyms): “very low calorie diet”, “very low calorie ketogenic diet”, “very low carbohydrate diet”, and “gut microbiota”. Our search strategy retrieved nine eligible studies. Overall, VLCDs and VLCarbDs affected the Bacteroidetes to Firmicutes ratio in obese patients, leading to a reduction in short-chain fatty acid production by fecal microbiota associated with Clostridial cluster XIVa. This reduction particularly affected *Roseburia* and *Eubacterium rectale*, the two most abundant butyrate-producing bacteria in human feces. VLCKDs preserved the core fecal microbiome, but altered the composition of fecal microbial populations in relation to the plasma metabolome and fecal bile acid composition. In particular, VLCKD-induced weight loss resulted in a reduction in *E. rectale* and *Roseburia*, an increase in *Christensenellaceae* and *Akkermansia* while not all studies show a decrease in *Faecalibacterium prausnitzii*. Although very few studies have analyzed the effects of VLCarbDs and VLCDs on gut microbiota, significant diet-induced changes in fecal microbiota composition have been observed. Further studies are needed.

## Introduction

The gut microbiota has been recognized as a key factor driving metabolic diseases such as diabetes, atherosclerotic cardiovascular disease, non-alcoholic fatty-liver disease, Alzheimer’s disease, and certain obesity-related cancers (1). It also plays an important role as a separate endocrine organ that maintains host energy homeostasis and stimulates host immunity through molecular crosstalk (2–4). Shifts in gut microbial composition due to extrinsic factors can significantly disrupt the symbiotic relationship between gut bacteria and the host, favoring the development of metabolic diseases.

Microbes in the human gut produce a wide range of metabolites, most of which are chemically similar to metabolites produced by host cells (e.g. nitric oxide, gamma-aminobutyric acid, serotonin, short-chain fatty acids [SCFAs], and indoles). Other metabolites, however, such as the bile acids, result from the chemical transformation of host molecules by microbes. Due to their similarity, these molecules are all recognized by host cells, and may act on specific receptors or trigger the release of other hormonal signals, such as the gut peptides glucagon-like peptide-1 and peptide YY, which both act on energy metabolism. Translocation of lipopolysaccharides (LPS) into the bloodstream can trigger low-grade inflammation affecting the liver and adipose tissue and altering muscle metabolism; it is an important hallmark of obesity, diabetes, and related disorders. In addition, those endotoxins can disrupt appetite regulation by altering the activity of the enteric nervous system and the gut-brain axis *via* the vagus nerve (4). The most common organisms in the human gut are members of the gram-positive Firmicutes phylum, which includes the genera *Eubacterium, Clostridium, Ruminococcus, Butyrivibrio*, and *Lactobacillus*; the gram-negative Bacteroidetes phylum, with the genera *Bacteroides, Prevotella*, and *Porphyromonas;* and the less abundant Proteobacteria, with the genus *Enterobacteria* (e.g. *Escherichia coli*); *Verrucomicrobiaceae*, with *Akkermansia municiphila*; and Actinobacteria, with the genus *Bifidobacterium* (5–7 *).* The human gut contains three main enterotypes dominated by *Ruminococcus, Bacteroides*, and *Prevotella*. Each enterotype uses a different energy pathway, and it has been hypothesized that the predominance of one genus over another is determined by the host’s diet (8). In addition, the composition of gut microbiota may be altered by certain diseases; it has been shown, for example, that the gut microbiota of people with diabetes has a different composition to that of healthy individuals (9). Diabetes and obesity are both characterized by low-grade inflammation of unknown molecular origin. A study by Cani et al. (10) showed that metabolic endotoxemia influences inflammatory tone, weight gain, and diabetes, and that high-fat nutrition modulates gut microbiota and plasma concentrations of LPS. Changes observed in the gut microbiota following antibiotic treatment suggest that these microorganisms might be involved in modulating metabolic endotoxemia, low-grade inflammation, obesity, and type 2 diabetes. This would also provide an explanation for some of the mechanisms involved (11).

Very low calorie diets (VLCDs) (high-protein, low-carbohydrate [ ≤ 30%] diets with a maximum intake of 800 kcal/day) and very low calorie ketogenic diets (VLCKDs) (<800 kcal/day, carbohydrates <70 g/day) are used in interventions designed to reduce body weight and improve fasting blood glucose, insulin, and lipid levels (12). VLCKDs induce ketosis, which is a metabolic state characterized by an increased concentration of ketones (acetoacetate, 3-β-hydroxybutyrate, and acetone) in the blood due to raised levels of fatty acid breakdown and ketogenic enzyme activity (13). The permitted daily intake is 70-100 g protein (or 0.8-1.5 g protein/kg of ideal body weight), <50 g of carbohydrates from vegetables, and 10 g of olive oil (14). VLCKDs involve the complete replacement of regular meals with food or formulations used under medical supervision for weight loss in individuals with a body mass index (BMI) >30 (or >27 if the person has obesity-related comorbidities) and in those requiring rapid weight loss (15). VLCDs are typically used as part of an intervention combining medical supervision and lifestyle changes. Under these conditions, they are generally considered safe and effective (14–16) and are gaining traction as a means of achieving rapid weight loss.

The aim of this review was to examine the potential effects of very low calorie diets (VLCDs), very low calorie ketogenic diets (VLCKDs) and very low carbohydrate diets (VLCarbDs) on the composition of the gut microbiota. To date, the studies on this topic in literature are scarce. Since gut microbiota is involved with the quantity and quality of nutrients extracted from the diets, with direct implications for obesity and related disorders, it’s interesting to investigate how VLCDs and VLCKDs affect the composition of the gut microbiota, and whether these modifications may be effective against obesity, diabetes, and related disorders.

## Materials and Methods

The present systematic review was performed following the steps by Egger et al. as follows (17): (1) A working group was configured as follows: three operators skilled in clinical nutrition, of whom one acting as a methodological operator and two participating as clinical operators. (2) The revision question on the basis of considerations made in the abstract was formulated as follows: “the state of the art of the effects of energy-restricted diets, namely very low calorie diet (VLCD), very low calorie ketogenic diet (VLCKD), and very low carbohydrate diet (VLCarbD), on the composition of the gut microbiota in humans.” (3) Relevant studies were identified as follows: a research strategy was planned, on PubMed, Cochrane Central Register of Controlled Trials (CENTRAL) and Web of Science as follows: (a) definition of the key words (Very low carbohydrate diet (VLCarbD), Very low calorie diet (VLCD), obesity, microbiota, Very low calorie ketogenic diet (VLCKD), gut microbiota), allowing the definition of the interest field of the documents to be searched, grouped in quotation marks (“…”), and used separately or in combination; (b) use of the Boolean AND operator that allows the establishment of logical relations among concepts; (c) research modalities: advanced search; (d) limits: time limits: papers published in the last 15 years; languages: English; (e) manual search performed by the senior researchers experienced in clinical nutrition through revision of reviews and individual articles on the effects of VLCD, VLCKD, and VLCarbD, on the composition of the gut microbiota in humans in journals qualified in the Index Medicus. (4) The analysis was carried out in the form of a systematic review of the reports.

## Results

The research has been carried out based on the following keywords: “very low calorie diet” OR “VLCD” OR “very low calorie ketogenic diet” OR “VLCKD” AND “gut microbiota”; This search retrieved six eligible studies, shown in **Table 1**. Three, of the six studies, are randomized controlled trials (18–20), one prospective cohort study (21), one controlled parallel design trial (22) end one randomized, single blind, parallel design trial (23). The second research has been based on: “very low carbohydrate diets” OR “VLCarbDs” AND “gut microbiota”. This search strategy retrieved three eligible studies, all randomized controlled trial, summarized in **Table 2** (24–26).

**Table 1:** Studies on the effects of VLCDs on gut microbiota.

AUTHOR, YEAR	TYPE OF STUDY	TYPE OF DIET	SAMPLE	DURATION	FINDINGS
**Simoes, 2014** (18)	Randomized trial	VLCD (VLED)	16 obese patients	12 months	Changes in fecal microbial numbers in obese individuals were primarily affected by dietary intake rather than by weight change. *Bifidobacteria* and *Lactobacillus* decreased after the VLED, but the change was transient.
		800 kcal, CHO 67 g, PROT 90 g, FAT 9.5 g			
**Sandrine, 2016** (19)	Randomized trial	VLCKD (VLCD)	16 obese patients	52 weeks	Reduction in the Bacteroidetes : Firmicutes ratio was related to metabolic syndrome rather than BMI. VLCD was associated with a reduction in *Roseburia* and an increase in *Akkermansia*. *Bifidobacteria* numbers did not fall, possibly due to the presence of inulin.
		(800 Kcal, CH0 <30%)			
**Basciani, 2020** (20)	Randomized controlled trial	VLCKD (VLCD)	48 obese patients	45 days	After diet, the relative abundance of Firmicutes was significantly decreased while Bacteroidetes increased proportionally with the only exception in the VPG in which the increase in Bacteroidetes not reached statistical significance suggesting that the origin of proteins may influence the microbiota change
		90 g protein	divided in three group: 1- WPG 2- VPG APG		
		26 g carbohydrates			
		15 g lipids			
**Aleman, 2018** (21)	Prospective cohort study	VLCKD (VLCD)	1- 10 obese postmenopausal women	46 days	*Roseburia* decreased and *Christensenellenaceae* increased after VLCKD. No significant changes were observed in the Bacteroidetes : Firmicutes ratio. During ketosis, β-hydroxybutyrate production was negatively correlated with *F. prausnitzii* and *Roseburia* contrasting with the results of other studies cited above. The question remains to be explored.
		54% PROT			
		26% CHO			
		20% FAT			

**Carolina Guitérrez-Repiso, 2021** (22)	Controlled parallel design trial	VLCKD (VLCD)	61 obese patients divided in three group: VLCKD (18) MetDiet (21) BS (22)	2 months	In this study, as in other previous ones, in patients who underwent VLCKD there was a significant increase in Alistipes (Rikenellaceae family) while a decrease in Lactobacillus was recorded. There was also a decrease in Orodibacter splanchnicus (which in the previous study by the same author only increased if it was accompanied by probiotic supplementation) and there was an increase of Parabacteroides.
**Guitérrez-Repiso, 2019** (23)	Randomized, single blind, parallel-design	VLCKD (VLCD)	33 obese patients divided in three group: 2- synbiotics1-sunbiotc2, 3- placebo-synbiotic2, control.	2 months (VLCKD) + 2 months (LCD)	The authors verify that the VLCKD program not alter the gut microbial population and that the Bacteroidetes/Firmicutes ratio correlates significantly with the percentage of weight loss. In particular, it is evident, in the placebo/synbiotc2 group, that the administration of probiotics such as *Bifidobacterium animal subps lactis* and prebiotic fibers was able to increase the weight gain (compared to the control) also decreasing the inflammatory state.
		75 g protein			
		20 g carbohydrates			
		3 g fat			

BMI, body mass index; CHO, carbohydrates; VLCD, very low calorie diet; VLCKD, very low calorie ketogenic diet; VLED, very low energy diet; PROT, proteins; WPG, diet with whey protein group; VPG, diet with vegetable protein group; APG, diet with animal protein group; MetDiet, Mediterranean Diet, BS, Bariatric Surgery.

**Table 2:** Studies on the effects of VLCarbDs on gut microbiota.

AUTHOR, YEAR	TYPE OF STUDY	TYPE OF DIET	SAMPLE	DURATION	FINDINGS
**Duncan Sylvia H et al., 2007** (24)	Randomized trial	VLCarbD (HPLC) 30% PROTEIN 4% CHO 66% FAT	19 obese individuals	9 weeks	HPLC diet led to reductions in *E.rectale, Roseburia*, and SFCAs
**Ley RE at al., 2006** (25)	Randomized trial	VLCarbD (CARB-R)	12 obese individuals	52 weeks	CARB-R diets led to an increase in Bacteroidetes and a decrease in Firmicutes, increasing the Bacteroidetes: Firmicutes ratio, which was lower in obese patients than in healthy individuals.
**Duncan Sylvia H et al., 2008** (26)	Randomized trial	VLCarbD (LC)	23 obese individuals	8 weeks	LC diet led to reductions in Firmicutes *(Roseburia + E.rectale)*

CARB-R, charbohydrate – restricted; CHO, carbohydrate; HPLC, high-protein/low-carbohydrate; LC, high-protein low carbohydrate ketogenic; SCFAs, short-chain fatty acids; VLCarbD, very low-carbohydrate diet.

**Figure 1:**
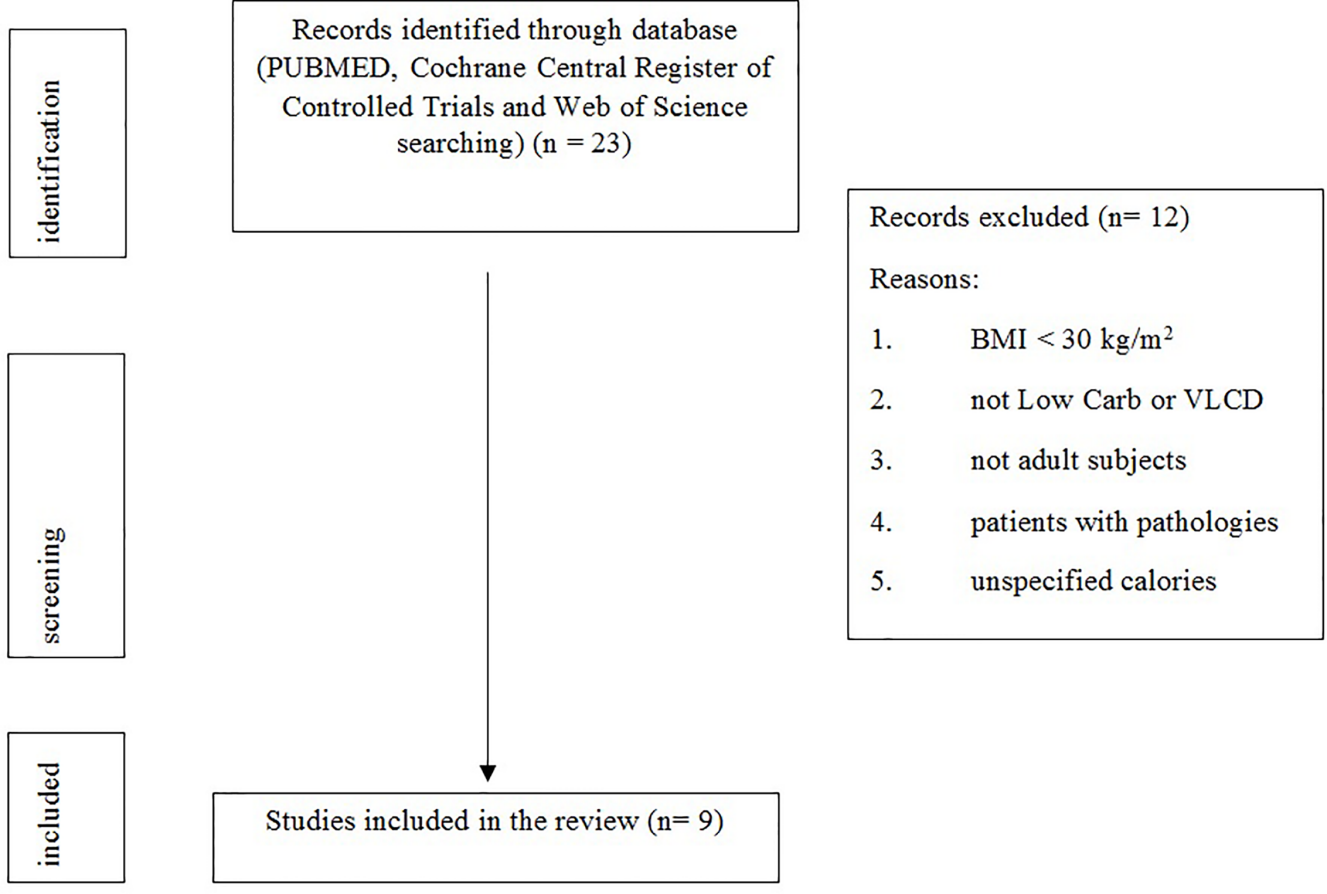
Flow diagram of the review process.

**Figure 1** shows the study selection process.

## Discussion

The human gut contains a complex community of microbes that have a pivotal role in human health. It is estimated to contain around 1000 bacterial species and 100 times more genes than the human genome (27). This community of microbes is often referred to as a “hidden metabolic organ” because of its enormous influence on host metabolism, physiology, nutrition, and immune function (28).

Any dietary changes (e.g., FODMAPs, gluten free or VLCDs), despite showing beneficial effects, can affect microbiota composition, especially when protracted for a long time (29).

During a weight loss program, the relative abundance of Bacteroidetes increased and the abundance of Firmicutes decreased irrespective of diet type as long as the person lost at least 6% of their body weight (20, 25).

VLCarbDs and VLCDs appeared to reduce the abundance of just a few groups of bacteria with health benefits, and they were also associated with increased proportions of others, such as *A. municiphila* and *Christensenella*. The increased proportions of bacteria, such as *Akkermansia* and *Christensenella*, induced by a low-calorie intake, represents an intriguing example of host-microbe co-evolution that would appear to benefit both partners (6, 30, 31). *Akkermansia muciniphila* is inversely correlated with disease status (32). *Akkermansia muciniphila*, is a mucin-degrading bacterium that resides in the mucus layer. The presence of this bacterium inversely correlates with body weight in rodents and humans. However, the precise physiological roles played by this bacterium during obesity and metabolic disorders are unknown (33).

Low carbohydrates intake in very low energy diet (VLED) reduced substrates available for *Bifidobacteria* and bacteria within the *Lactobacillus* group in the large intestine, while the high content of protein contributed to *Bacteroides spp* increase (18), the predominant proteolytic species identified in the human large intestine (34). In maintenance phase, at the end of VLED, *Bifidobacteria* and bacteria within the *Lactobacillus* group start to increase again suggesting that change is transient. Drastic dietary changes in VLED leads so to *Bacteroides* spp. increase and to *Bifidobacteria* decrease, reflecting that bacteria flora modifications are associated with dietary intake rather the body weight variations (18).

Duncan et al. investigated the effects on gut microbiota of two diets, high-protein low carbohydrate, ketogenic (LC) and high-protein moderate-carbohydrate, non-ketogenic (MC). Impact of each diet on gut microbiota was very different: particularly the LC diet led to decrease of Firmicutes (*Roseburia* and *Eubacterium rectale*) some of which are responsible for butyrate production (26).

Similarly, Russell et al. observed that the populations of the butyrate producing *Roseburia/E. rectale* group of bacteria decreased markedly with the high protein and low carbohydrate (HPLC) normocaloric diet. The abundance of another group of butyrate producers is maintained, which are related to *Faecalibacterium prausnitzii* (35). This group may have become the main supplier of butyrate in the intestine with this diet. Butyrate is the main source of energy for the colon cells; therefore, it allows cells to replicate and function normally, avoiding the apoptosis. *Faecalibacterium prausnitzii* exercises beneficial anti-inflammatory effects on the intestinal mucosa (36) and it is believed to have a positive influence on colon health (35).

The bacteria that are most affected by the decrease in carbohydrate intake are *E. rectale* and *Roseburia* while *F. prausnitzii* seems to be less affected by the decrease in carbohydrates in the diet. Duncan et al., monitored dietary effect shift from normal intakes of carbohydrate (399g/day) to either moderate (164g/day) or low (24g/day) intakes in a weight loss program for obese individuals, showing a *E. rectale* and Roseburia spp. marked progressive decrease with reduction of carbohydrate intake (24).

*Roseburia* and *E. rectale* belong to the Firmicutes phylum and they are part of the commensal bacteria that produce short chain fatty acids (SCFA), in particular butyrate, which influence colon motility, immunity maintenance and anti-inflammatory properties. Changes in the presence of *Roseburia* can lead to the development of various diseases (including irritable bowel syndrome, obesity, type 2 diabetes, nervous system conditions and allergies) (37).

*Faecalibacterium prausnitzii*, an anerobic bacterium belonging to the Firmicutes phylum, is a key component of the gut microbiota and the most abundant butyrate-producing bacterium in the human colon (38). It accounts for approximately 5% of all detectable bacteria in stool samples from healthy adults (39). *Faecalibacterium prausnitzii* is best known as a biomarker for human health, as decreased levels have been associated with increased inflammatory activity and may trigger certain diseases such as colorectal cancer (38). Reduced numbers have been reported in inflammatory bowel disease and infectious colitis, and Furet et al. (40) showed that this species might also play a role in low-grade inflammatory disorders such as obesity and diabetes. The authors also observed a relationship between *F. prausnitzii* and inflammatory markers in both non-diabetic and diabetic obese patients, even after adjustment for BMI. *Faecalibacterium prausnitzii* proportions were smaller in those patients with type 2 diabetes presenting higher levels of low-grade inflammation and insulin resistance. A negative association was also seen between *F. prausnitzii* and insulin resistance assessed using the homeostatic model assessment for insulin resistance (HOMA IR), possibly attributable to an improvement in glucose metabolism in the diabetic group (40).

In contrast to the previous studies, Aleman et al., observed that during a VLCKD the increased lipolysis leaded to production of β-hydroxybutyrate (BHB), a ketone body that is also a short chain fatty acid byproduct, that resulted negatively correlated with *F. prausnitzii* and genus *Roseburia.* The increase of BHB levels could results from less catabolism by *F. prausnitzii* and *Roseburia* (21).

Further studies on VLCKD were carried out between 2019 and 2021. Guiterrez Repiso et al. revealed that the VLCKD program not alter the gut microbial population and that the Bacteroidetes*/*Firmicutes ratio correlates significantly with the percentage of weight loss (23). VLCKD positively changes the microbiota but this change is greater if probiotics and prebiotics are supplemented during the diet (23).

A recent study by Basciani et al. showed that the microbiota is sensitive to the type of proteins of the diet. Patients were administered with a VLCKDs with whey, vegetable or animal proteins; the relative abundance of Firmicutes was significantly decreased while Bacteroidetes increased proportionally with the only exception in the vegetable protein group in which the increase in Bacteroidetes not reached statistical significance suggesting that the origin of proteins may influence the microbiota change (20).

More recently Guitérrez-Repiso et al. compared the effects on the intestinal microbiota in patients following the Mediterranean diet (MetDiet), VLCKD, and who underwent bariatric surgery (BS) (22).

In patients administered with VLCKD there was a significant increase in *Alistipes* (Rikenellaceae family) while a decrease in *Lactobacillus* was recorded. There was also a decrease in *Orodibacter splanchnicu*s and there was an increase of *Parabacteroides. Alistipes* and *Parabacteroides* were negatively associated with waistline and body mass index in adults (41) and young people (42).

The gut microbiota is a very dynamic entity influenced by nutritional behaviors (43). The composition of the gut microbiota differs in obese and lean subjects, suggesting that microbiota dysbiosis can contribute to changes in body weight (44). While nutritional status (normal weight, overweight) is known to influence the microbial population, there are also studies that have evaluated the influence of the changes in microbiota on the pathways related to obesity and weight loss. In fact, the Firmicutes phylum has been shown to be negatively correlated with the resting energy expenditure (REE) as well as positively correlated with fat mass percentage (45). Moreover, a 20% increase in the Firmicutes phylum abundance was associated with an increase of 150 kcal in energy harvest (46). On the other hand, a decrease in the Firmicutes-to-Bacteroidetes ratio after a weight loss program also was observed, and the Bacteroidetes proportion was positively correlated with a percentage of loss of body fat (25). In addition, it was observed that the persistent success after a VLCKD (no weight recover in the following 2 years) was associated with the microbiota; the *Alistipes*, a Bacteroidetes member of Rikenellaceae family was associated with program success, while *Prevotella* abundance was associated with less success (24).

Several papers investigated the gut microbiota correlation with diet and weight loss, but studies on the effects of specific VLCDs, VLCKDs and VLCarbDs on the microbiota are scarce and the results are controversial. The strength of the study is to selectively investigate the available literature on the effect of carbohydrate-restricted diet (VLCDs, VLCKDs and VLCarbDs) on the changes in the intestinal microbiota.

## Conclusions

Although very few studies have analyzed the effects of VLCarbDs and VLCDs on gut microbiota, significant diet-induced changes in fecal microbiota composition have been observed. Further studies are needed.

## Author Contributions

MR designed and wrote the paper; CG, GP, SP, PB, IS, RM and CR wrote and edited the paper, MF and MN visualized the paper. All authors contributed to the article and approved the submitted version.

## Conflict of Interest

PB, IS and RM were employed by company PronoKal Group.

The remaining authors declare that the research was conducted in the absence of any commercial or financial relationships that could be construed as a potential conflict of interest.
