# Corrigendum: The Potential Roles of Very Low Calorie, Very Low Calorie Ketogenic Diets and Very Low Carbohydrate Diets on the Gut Microbiota Composition

**DOI:** 10.3389/fendo.2021.825790

**Published:** 2021-12-22

**Authors:** Mariangela Rondanelli, Clara Gasparri, Gabriella Peroni, Milena Anna Faliva, Maurizio Naso, Simone Perna, Philip Bazire, Ignacio Sajoux, Roberto Maugeri, Chiara Rigon

**Affiliations:** ^1^ IRCCS Mondino Foundation, Pavia, Italy; ^2^ Department of Public Health, Experimental and Forensic Medicine, Unit of Human and Clinical Nutrition, University of Pavia, Pavia, Italy; ^3^ Endocrinology and Nutrition Unit, Azienda di Servizi alla Persona “Istituto Santa Margherita”, University of Pavia, Pavia, Italy; ^4^ Department of Biology, College of Science, University of Bahrain, Sakhir, Bahrain; ^5^ PronoKal, London, United Kingdom; ^6^ PronoKal Group, Barcelona, Spain; ^7^ PronoKal Group, Savigliano, Italy

**Keywords:** very low carbohydrate diet (VLCarbD), very low calorie diet (VLCD), obesity, microbiota, very low calorie ketogenic diet (VLCKD), gut microbiota

In the article as originally published, the name of the eighth author was incorrectly spelled as “Ignacio Sajuox”. The correct spelling is “Ignacio Sajoux”.

The authors apologize for this error and state that this does not change the scientific conclusions of the article in any way. The original article has been updated.

## Publisher’s Note

All claims expressed in this article are solely those of the authors and do not necessarily represent those of their affiliated organizations, or those of the publisher, the editors and the reviewers. Any product that may be evaluated in this article, or claim that may be made by its manufacturer, is not guaranteed or endorsed by the publisher.

